# Admission of alumni from Multiprofessional Residency Programs into the SUS

**DOI:** 10.11606/s1518-8787.2021055003347

**Published:** 2021-11-23

**Authors:** Taiana Brito Menêzes Flor, Nirond Moura Miranda, Cristiane da Silva Ramos Marinho, Josilene Maria Ferreira Pinheiro, Pedro Henrique Sette-de-Souza, Luiz Roberto Augusto Noro

**Affiliations:** I Universidade Federal do Rio Grande do Norte Programa de Pós-Graduação em Saúde Coletiva Natal RN Brasil Universidade Federal do Rio Grande do Norte. Programa de Pós-Graduação em Saúde Coletiva. Natal, RN, Brasil; II Universidade Federal do Rio Grande do Norte Curso de Graduação em Odontologia Natal RN Brasil Universidade Federal do Rio Grande do Norte. Curso de Graduação em Odontologia. Natal, RN, Brasil; III Universidade de Pernambuco Faculdade de Odontologia Arcoverde PE Brasil Universidade de Pernambuco. Faculdade de Odontologia. Arcoverde, PE, Brasil; IV Universidade Federal do Rio Grande do Norte Centro de Ciências da Saúde Departamento de Odontologia Natal RN Brasil Universidade Federal do Rio Grande do Norte. Centro de Ciências da Saúde. Departamento de Odontologia. Natal, RN, Brasil

**Keywords:** Patient Care Team, Non-medical Boarding School, Work Status, Primary Health Care, Unified Health System

## Abstract

**INTRODUCTION:**

The Multiprofessional Health Residency Programs (PRMS) were set up as a strategy for training workforce for the Brazilian Unified Health System (SUS).

**OBJECTIVE:**

To investigate the proportion of alumni from Primary Health Care Multiprofessional Residency Programs admitted into the SUS and associated factors.

**METHODS:**

This is a sectional study developed with alumni from Primary Health Care Multiprofessional Residency Programs from all over Brazil, encompassing the period from 2015 to 2019. Participants answered an online questionnaire with general personal information, admission into *stricto sensu* graduate school, the labor market and, specifically, the SUS. We applied Pearson's chi-square test for bivariate analyses and Poisson's regression for multiple analysis.

**RESULTS:**

A total of 365 alumni from Programs from all Brazilian regions participated in the study. Of those, 80.2% reported entry into the labor market and 47.9% reported being employed in the SUS. Admission into the SUS has been associated with the professions that make up the Reference Team for Primary Health Care (PHC) (PR = 1.87; 95% CI 1.54–2.28) and non-admission into *stricto sensu* graduate programs (PR = 0.77; 95% CI 0.61–0.97). Regarding admission characteristics, the PHC scenario (47.4%) and work focused on health care (84.9%) were prevalent. Almost 40% of alumni who entered the SUS are working with unstable contracts. Besides, being a residency alumnus is often undervalued in recruitment (56.9%). Among those admitted into the SUS, 8.7% reported being selected to work in the Covid-19 pandemic effort.

**CONCLUSIONS:**

The results of this study reinforce the need for a policy to encourage the maintenance, creation and valorization of the PRMS. They also warn about the possibility that admission into the SUS for workers is increasingly difficult due to the current underfunding of the health system.

## INTRODUCTION

The health care model has undergone changes over the years, shifting from a biological perspective to a comprehensive health care approach. Based on this reality, the Unified Health System (SUS) requires professionals trained for the health demands of the population, with practices aimed at interventions on the social determinants of the health-disease process, through actions for disease prevention and propagation of health.

Especially since the 2000s, several initiatives have emerged with the goal of bringing health training closer to the needs of the SUS. Prominent actions in this context include the publication of the National Curricular Guidelines since 2001^1^, and inductive actions such as the National Program for Reorientation of Professional Health Training (*Pró-Saúde*) and the Health Care Work Training Program (*PET-Saúde*)^2^. Despite these initiatives, the strategies devised by most of the undergraduate programs are insufficient to overcome the obvious limitations and discrepancies between health training and the needs of the SUS^3^.

In this sense, the Multiprofessional Health Residency Programs (PRMS) are presented as *latu sensu* graduate opportunities created with the goal of training professionals for the SUS, focusing the work as a guiding element of training^4-6^. The programs are aimed at health care professions, with the exception of the medical profession, and aim to “promote the qualified admission of young health professionals into the labor market, particularly in priority areas of the SUS” (p. 2)^7^.

At the same time as the initiatives for changes in health care education, the provision and settlement of health professionals has been a concern in Brazil, especially in Primary Health Care (PHC). In the last decade, notable actions are the Program for Valorization of Primary Care (PROVAB), aimed at doctors, nurses and dentists, and the Program More Doctors for Brazil (PMM), aimed at the medical profession. Both focus on providing professionals for remote and vulnerable areas^8^. However, contrary to all the aforementioned stimuli, growing austerity policies in Brazil are causing the defunding of the health system.

Given this situation, it is important to investigate the admission of PRMS alumni into the SUS, which would mean a return on the investment in this type of education for society. The subject becomes especially relevant due to the lack of studies in this line, or studies showing that training in PMRS is an advantage for being admitted into the SUS. Therefore, given the situation presented, the objective of this study was to investigate the admission of alumni from PRMS aimed at PHC in the labor market, especially in the SUS, and also to identify possible associated factors.

## METHODS

This study is an excerpt from the research entitled “*Impacto da inserção de egressos das Residências Multiprofissionais no desenvolvimento do Sistema Único de Saúde*”. This is a sectional study conducted with alumni from PMRS focused on PHC from all over Brazil. The inclusion criteria used were the affiliation of the program to public higher education institutions, public health schools or government schools; being in operation in 2019; and having alumni from the period of 2015 to 2019.

Prior to sending the invitations to the institutions, we requested the list of PMRS in operation to the Ministry of Education, in May 2019, through request number 23480009406201916 in the Electronic System of the Citizen Information Service. With this information, we identified the programs that had one of the following terms in their names: “Primary Care”, “Family Health” or “Family and Community Health”. After prior contact 22 programs of the 37 eligible agreed to participate in the study.

**Figure 1:**
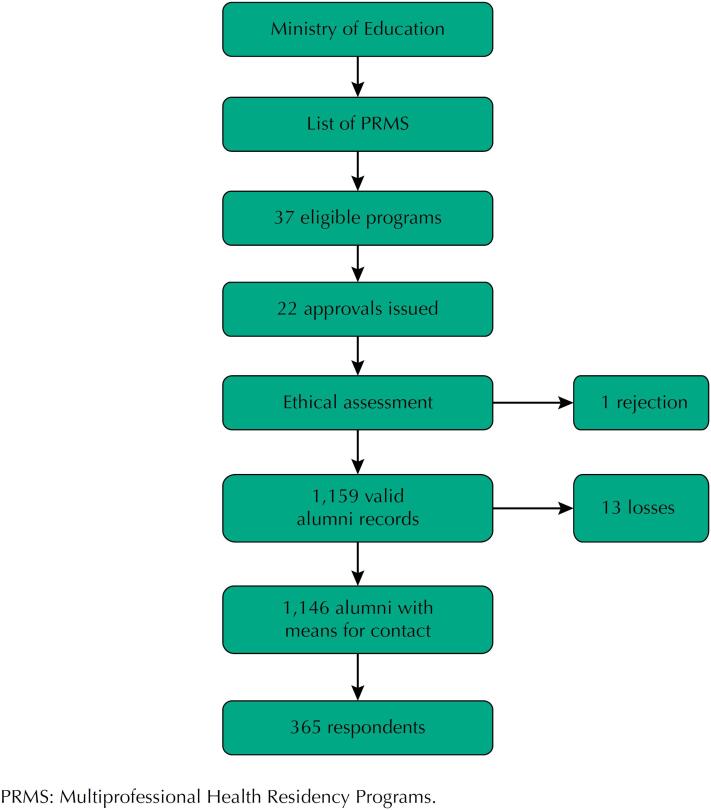
General flow chart of the study.

The project and its amendments were evaluated by the Research Ethics Committee of *Hospital Universitário Onofre Lopes* (CEP/HUOL), with favorable opinions numbers 3,744,514, 3,829,247 (Amendment 1) and 3,898,156 (Amendment 2). In addition, the project was processed by the research ethics committees of all the participating institutions that had such a committee, with 1 refusal. Figure 1 illustrates the overall flowchart of the study.

When the relevant ethical approvals were presented, each institution was asked to provide a list of names of alumni from the respective programs, with their e-mail addresses and telephone numbers. Dropouts, disconnected or dismissed students participating in the programs in the aforementioned period (2015–2019) were excluded of this study.

We then identified 1,159 records of valid alumni, but not all of the records had all the requested data (e-mail address and telephone). The alumni were invited to participate in the study mostly by email: an invitation and up to five reminders in a decreasing space of days between them were sent. The first reminder was sent five days after the invitation, the second after four days, and so on. In cases where the e-mail address was invalid, we searched the internet for another valid address or social networks of the alumni. When the search for a valid email was unsuccessful, the invitation was sent via social networks or messaging application in cases where the mobile phone number was up-to-date. Data collection took place in the period from June 1, 2020 to September 1, 2020.

A sample of 289 individuals within a total of 1,159 alumni would be sufficient for this study, considering a categorical outcome, 95% confidence interval, and 5% margin of error. However, the number of respondents we obtained was higher than necessary.

The data were collected with a self-applied online questionnaire sent using the Google Forms application. To access the questionnaire, alumni were asked to agree to participate in the study by an electronic means after having access to the objective of the study, its risks and benefits, and the informed consent form. The collection instrument was organized in closed or mixed questions addressing: 1 – general personal information and college education; 2 – aspects regarding the other graduate program; 3 – admission into the labor market; 4 – admission into the SUS; 5 – training in the PRMS, including the dimensions “personal motivation”, “pedagogical approach” and “in-service education scenarios”. The matrix of evaluation criteria proposed in item five underwent prior validation using the Delphi consensus technique.

We defined the outcome variable as the proportion of professionals working in the SUS and its characteristics (manner of selection, recognition of residency in the selection process, work relationship, time spent in admission, level of care, activities performed, contribution of residency to work and admission in view of the Covid-19 pandemic). Independent variables were defined as age, gender, skin color, profession, place of residency and admission into *stricto sensu* graduate programs, past or present. All are categorical variables.

We used Pearson's chi-square test for the bivariate analyses and Poisson's regression for multiple analysis, with robust variance. We included in the model independent variables with p-value ≤ 0.20 in the bivariate analysis. Those with significance ≤ 0.05 remained in the final model. The proposed model met the assumptions of significance of the Omnibus Test, absence of super-dispersion and adjustment of the model. For the inferential analyses, the variable “profession” was recategorized in Reference Team for Primary Care (Nursing and Dentistry) and Support Team (other professions), according to Campos^9^. All analyses were performed with the aid of the IBM SPSS version 20 statistical software.

## RESULTS

Of the 1,159 alumni who met the eligibility criteria, 13 were discarded due to failure to reach them by one of the described means. In all, 365 alumni from programs installed in all Brazilian regions participated in the study. The proportion of respondents by region where they attended residency matches the proportions of the records received (Table 1). In addition, in each region at least 40% of Primary Health Care Multiprofessional Residency Programs in operation participated in the study. Of all the participants, 80.2% declared that they have started working, with or without a connection to the SUS. According to the data available in Table 1, it can be seen that nationwide admission of workers into the SUS amounted to 47.9% (n = 175). Comparing the admission into the SUS amongst the Brazilian regions, we find a higher admission rate in the Southeast and Northeast regions, as both have higher rates than the national rate (Table 1). We also highlight that, of the total number of participants who reported admission into the SUS (n = 175), 35.4% (n = 62) also pursue activities in other services (or activities), with no connection to the SUS.

**Table 1:** Distribution of the number of records of alumni received, the number of study participants and professional admission into the Unified Health System (SUS) according to the region of Brazil where the residency program was completed, Brazil, 2020.

	Records		Respondents		Admission into the SUS	
	n	%	n	%	n	%
North	74	6.4	21	5.8	7	33.3
Northeast	837	72.2	263	72.1	135	51.3
Midwest	32	2.8	11	3.0	3	27.3
Southeast	136	11.7	37	10.1	22	59.5
South	80	6.9	33	9.0	8	24.2
Brazil	1,159	100.0	365	100.0	175	47.9

**Figure 2:**
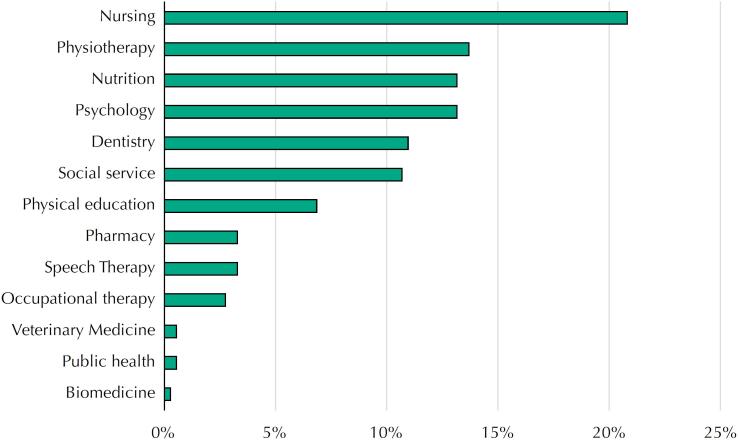
Distribution of alumni respondents from Multiprofessional Residency Programs in Primary Care by profession, Brazil, 2020.

There was also a predominance of people under the age of 30 years (54.2%), self-identified as female (81.8%) and self-declared as brown, black or Indigenous (56.7%) (Table 2). Of the thirteen professions indicated, the most frequent ones were as follows: nursing (20.8%), physiotherapy (13.7%) and nutrition and psychology, both with 13.2% of the answers (Figure 2). Regarding admission into the SUS (Table 2), we noted a significant association to this outcome, both in the bivariate analysis and the multiple model, of professions that make up the Reference Team (adjusted PR = 1.87; 95% CI 1.54–2.28) and admission into *stricto sensu* graduate school. The latter was seen as a factor that reduces the frequency of the outcome (adjusted PR = 0.77; 95% CI 0.61–0.97). If these two conditions are kept, the model predicts an increase in the estimated frequency of the outcome to 76%.

**Table 2:** Professional admission into the Unified Health System, according to the characteristics of alumni from Multiprofessional Residency Programs in Primary Care, Brazil, 2020 (n = 365).

Variables		Total		Admission into the SUS				Not adjusted		Adjusted	
				Yes		No					
		n	%	n	%	n	%	p^a^	PR (95% CI)^b^	p^c^	PR (95% CI)
Age (years)											
	< 30	198	54.2	91	46.0	107	54.0	0.408	1		
	≥ 30	167	45.8	84	50.3	83	49.7		1.09 (0.88–1.36)	–	–
Gender											
	Female	297	81.8	144	48.5	153	51.5	0.824	1.03 (0.78–1.37)	–	–
	Male	66	18.2	31	47.0	35	43.0		1		
Color											
	White and yellow	157	43.3	69	43.9	88	56.1	0.156	1		
	Black, brown and indigenous	206	56.7	106	51.5	100	48.5		1.17 (0.94–1.46)	–	–
Profession											
	Reference team	116	31.8	81	69.8	35	30.2	< 0.001	1.85 (1.52–2.26)	< 0.001	1.87 (1.54–2.28)
	Support team	249	68.2	94	37.8	155	62.2		1		
Place of program											
	Capital city	104	28.5	51	49.0	53	51.0	0.792	1		
	Countryside	261	71.5	124	47.5	137	52.5		0.97 (0.77–1.22)	–	–
Admission of *stricto sensu* graduate alumni											
	Yes	125	34.2	51	40.8	74	59.2	0.049	0.79 (0.62–1.00)	0.030	0.77 (0.61–0.97)
	No	240	65.8	124	51.7	116	48.3		1		

PR: prevalence ratio; Reference team: Nursing and Dentistry; Support team: other professions.

a Pearson's chi-square test.

b Number 1 signals the reference category.

c Poisson's regression.

Table 3 presents the characteristics of admission into the SUS. In this regard, data show that although alumni undergo formal selection processes in most cases, part of them perform professional activities in the SUS with unstable work ties, such as temporary contracts (27.2%) and scholarships (8.1%). The most frequent manner of admission was admission into primary care alone (47.4%), although part of the participants (15%) had been admitted into more than one context. There is also a predominance of activities (or actions) in health care (84.9%) and health management (18.6%). Although the respondents broadly acknowledge the contribution of training for the work (97.1%), 56.9% of the responses suggest that the residency program is not given due recognition in selection processes for working in the SUS.

**Table 3:** Characteristics of professional admission of alumni from Primary Care Multiprofessional Residency Programs into the Unified Health System, Brazil, 2020.

Variables		n	%
Selection type (n = 173)			
	Public tender	73	42.2
	Selection process	64	37.0
	Hiring via cooperatives	13	7.5
	Appointed official	10	5.8
	Contract	9	5.2
	Other	4	2.3
Differentiation of title scores for specialization in residency format (n = 167)			
	Yes	72	43.1
	No	95	56.9
Employment relationship (n = 173)			
	Hired employee	32	18.5
	Civil servant	67	38.7
	Scholar	14	8.1
	Appointed official	9	5.2
	Temporary contract	47	27.2
	Other	4	2.3
Time spent between completion of residency and start of work in the SUS (n = 170)			
	Up to 6 months	107	62.9
	From 7 months to 1 year	22	12.9
	More than 1 year	41	24.1
Level of attention (n = 173)			
	Primary care	82	47.4
	Medium complexity	25	14.5
	High complexity	27	15.6
	Primary care and medium complexity	10	5.8
	Primary care and high complexity	6	3.5
	Medium complexity and high complexity	7	4.0
	Three levels of attention	3	1.7
	Other context	13	7.5
Characterization of the professional activity performed (n = 172)^a^			
	Health care	146	84.9
	Health management	32	18.6
	Health surveillance	21	12.2
	Health education	28	16.3
	Preceptorship	25	14.5
Perceived contribution of residency training for work (n = 172)			
	Yes	167	97.1
	No	5	2.9
Admission by selection for Covid-19 pandemic effort (n = 172)			
	Yes	15	8.7
	No	157	91.3

a Exhaustive categorical variable.

## DISCUSSION

The concern with the admission and performance of PRMS alumni in the SUS is not recent^10,11^. However, until the present there is little information in this regard. We found in the scientific literature a study conducted with alumni from the Medical Residency Program, but focusing on a single program^12^. National initiatives for studies involving the work performance of alumni were located within the scope of the public health undergraduate program^13^ and *stricto sensu* graduate programs in health care^14^. Lima and Andriola^15^ stress that alumni from undergraduate or postgraduate courses take on a strategic role for understanding the social and professional effectiveness of the training they undergo.

In this sense, the results presented make a great contribution to the scientific literature, especially considering the present moment, when, on the one hand, we experience threats and setbacks in the SUS and, on the other hand, we face the greatest challenge to health in the last 100 years due to the Covid-19 pandemic.

When we talk about the admission of health professionals into PHC, it is important to highlight that this incorporation takes place into one of the teams provided for in the National Primary Care Policy (PNAB)^16^, which are: Family Health Team (ESF), Primary Care Team (EAB), Oral Health Team (ESB), Extended Family Health and Primary Care Center (NASF-AB) and Community Health Agents Strategy. While the ESF, EAB and ESB provide opportunities for the admission of nurses and dentists (in addition to doctors), the NASF-AB allows the admission of several professions such as social workers, physical educators, pharmacists, physiotherapists, speech therapists, nutritionists, psychologists, occupational therapists, veterinarians, and public health specialists, in addition to some medical specialists^16^.

The NASF-AB are referenced on the matrix support methodology^17,18^, which enables the admission of specialists into Brazilian PHC by offering rearguard and technical-pedagogical support for the reference teams (ESF or EAB). It may become a standard shared care arrangement and raise the conclusion rates of Primary Care^17,19^. However, the maintenance of this device is threatened by Technical Note No. 3/2020, which unbinds the offer of multiprofessional team services to the NASF-AB team typology, ceasing the accreditation of new teams and providing the town manager with the choice of professionals of the multiprofessional team to be hired, their work hours and team arrangements^20^.

In this sense, based on the results presented in this study, it is possible that the admission of Support Team workers into the SUS will happen at a slower pace due to the consequences of the technical note mentioned. We thus fear, as a result, that the decrease or lack of specialists may lower the conclusion rates of PHC services.

It is important to stress that, among the professionals admitted into the SUS, around 40% develop their work activities with unstable employment ties. Considering that temporary contracts depend on a certain political economic situation to be renewed ^15^, such instability can contribute to workers having low performances, with weak employment relationships, besides discouraging future alumni from residency programs due to the uncertainty of admission into the SUS.

The austerity measures that the political context imposes at this time, such as the public spending cap put into force by Constitutional Amendment 95/2016^21^, in addition to the curtailment of labor and social security rights and the possibility of outsourcing of health services^22^, create a disheartening scenario for the findings of this study.

Moreover, recent changes in the PNAB have jeopardized the PHC model under construction in Brazil. Among the changes is the recomposition of the EAB that, among other characteristics, admits 10 hours as the minimum workload for workers, strengthening an action based on the “treat ‘em and street ‘em” approach^23^. Conversely, this workload reduction weakens the ties between workers and users, compromising the kind of knowledge about some demands that can only be identified when there is a bond of affection.

Simultaneous to the collapse of the health system, we should mention the administrative reform proposed by the federal government, with Constitutional Amendment Bill 32/2020, which will be enforced to new civil servants of the three levels of the federation and, in practice, jeopardizes a series of career rights^24^.

The second variable associated with admission into the SUS was past or present admission of alumni into *stricto sensu* graduate programs, which disadvantaged admission into the SUS. Effectively, this means that by being admitted into *stricto sensu* graduate programs (master's or doctorate), the graduate students distance themselves from the professional admission into the SUS. This outcome may occur due to situations such as the choice of full-time dedication to graduate school, the offer of scholarships in graduate school and/or investment in a future teaching career.

A study conducted with alumni from undergraduate Public Health courses in Brazil showed that full-time dedication to graduate school was the second most frequent reason for not entering the labor market at the time of the research^13^. Such data highlight the great relevance of proposals for professional master's degrees with critical methodologies for workers in the health system, in addition to the important role of distance education as a mediation strategy^25,26^. In pedagogical conceptions based on critical methodologies, the learning cycle is organized in the form of situation-problems originated from reality of students, which helps in the construction of works focused on meeting locoregional needs^26^. Ceccim and Pinto^27^ emphasize that the commitment to fight regional and social inequalities is part of the encounter of health care training and the health systems and services, presupposing organic relationships.

Though there is practically no doubt about the importance of PRMS for the work in the SUS, the alumni point out that this training is clearly undervalued in hiring processes for working in the SUS. This shows the relevance of building a National Policy of Health Residencies that provides guidelines for the valorization of PRMS alumni in public tenders, as well as qualification and better support of for the operation of the existing programs^28^.

Finally, we highlight the admission of part of the alumni into the SUS from selective processes aimed at fighting the Covid-19 pandemic. The pandemic profoundly affects health services, requiring expansion of the workforce, care delivery structures and inputs to sustain the effort against it^29^. Despite years of underfunding^21^ and the lack of nationwide coordination in coping with the pandemic^30^, it is the Brazilian National Health System that serves the majority of Brazilians and, to this end, demands professionals aligned with its challenges, principles and guidelines.

Limitations to this study are the unavailability of previous information about the admission of the respondents into the SUS, which would allow a more thorough understanding of their professional trajectories to date. This limitation is inherent to the study design adopted, which is capable of showing only one cross-section in time. In spite of this, we understand that the study makes an important contribution to the debate of training for the SUS, as it investigated the professional admission of alumni from Primary Health Care Multiprofessional Residency Programs admitted into the SUS and associated factors.

The effectiveness of PRMS in PHC was clearly identified in this study when it was found that approximately 80% of the alumni studied were admitted into the labor market. Regarding alumni admitted into the SUS, the study made the important discovery that such admission is unstable and flexible, materialized by the development of multiple activities and multiplicity of admission conditions. Another important point that we highlight is the relevant perception of the contribution of PHC residency programs to the performance of SUS activities. It was shown that almost half of the alumni studied have joined the SUS, but the findings of this study reinforce the need to propose a policy of valorization of the PRMS in the recruitment process for working in the SUS.

In addition, the results warn of a possible increase in the difficulty of admission of Support Team workers into PHC due to the clearance of the NASF-AB, with a likely impact on the conclusion rates of the SUS. Furthermore, the administrative reform under discussion can negatively impact civil service careers, increasing the existing instability. These are more recent events in a larger context of defunding of the health system and destabilization of labor relations, reinforcing the relevance of conducting studies in this line.

The path we followed recommends continued studies focusing on the professional admission of PRMS alumni, addressing other areas of concentration or specialty. Like the other studies with alumni cited in this study, digital research proved to be a powerful communication tool, allowing contact with people from all over Brazil.
