# Supplementary material for: Admission of alumni from Multiprofessional Residency Programs into the SUS
Source: Rev Saude Publica. 2021 Nov 23;55:88. doi: 10.11606/s1518-8787.2021055003347 (PMC8647992; doi:10.11606/s1518-8787.2021055003347)
Supplement: Translation

# Translation: Inserção de egressos de Programas de Residência Multiprofissional no SUS

**Authors:** Taiana Brito Menêzes Flor, Nirond Moura Miranda, Cristiane da Silva Ramos Marinho, Josilene Maria Ferreira Pinheiro, Pedro Henrique Sette-de-Souza, Luiz Roberto Augusto Noro

**Affiliations:** I Natal RN Brasil Universidade Federal do Rio Grande do Norte. Programa de Pós-Graduação em Saúde Coletiva. Natal, RN, Brasil; II Natal RN Brasil Universidade Federal do Rio Grande do Norte. Curso de Graduação em Odontologia. Natal, RN, Brasil; III Arcoverde PE Brasil Universidade de Pernambuco. Faculdade de Odontologia. Arcoverde, PE, Brasil; IV Natal RN Brasil Universidade Federal do Rio Grande do Norte. Centro de Ciências da Saúde. Departamento de Odontologia. Natal, RN, Brasil

**Keywords:** Equipe de Assistência ao Paciente, Internato não Médico, Status de Ocupação Laboral, Atenção Primária à Saúde, Sistema Único de Saúde

## Abstract

**INTRODUÇÃO:**

Os Programas de Residência Multiprofissional em Saúde (PRMS) configuram-se como estratégia para a formação de força de trabalho para o Sistema Único de Saúde (SUS).

**OBJETIVO:**

Investigar a proporção de egressos de PRMS voltados à Atenção Primária à Saúde (APS) inseridos no SUS e fatores associados.

**MÉTODOS:**

Trata-se de um estudo seccional desenvolvido com egressos de PRMS voltados à APS de todo o Brasil, referente ao período de 2015 a 2019. Os participantes responderam a um questionário *on-line* com informações pessoais gerais, inserção na pós-graduação *stricto sensu*, no mercado de trabalho e especificamente no SUS. Foram aplicados o teste qui-quadrado de Pearson, para análises bivariadas, e Regressão de Poisson, para a análise múltipla.

**RESULTADOS:**

Participaram do estudo 365 egressos de Programas de todas as regiões brasileiras. Destes, 80,2% relataram inserção no mercado de trabalho e 47,9% informaram estar trabalhando no SUS. A inserção no SUS esteve associada às profissões que compõem a Equipe de Referência para a APS (RP = 1,87; IC95% 1,54–2,28) e à não inserção em programas de pós-graduação *stricto sensu* (RP = 0,77; IC95% 0,61–0,97). Quanto às características de inserção, prevaleceram o cenário da APS (47,4%) e o trabalho voltado à atenção à saúde (84,9%). Quase 40% dos egressos inseridos no SUS estão trabalhando por intermédio de vínculos instáveis, além de frequente não valorização do título da residência no recrutamento (56,9%). Entre os inseridos no SUS, 8,7% relataram terem sido selecionados para o enfrentamento da pandemia de covid-19.

**CONCLUSÕES:**

Os resultados deste estudo reforçaram a necessidade de política de incentivo à manutenção, criação e valorização dos PRMS e alertaram para possível aumento da dificuldade de inserção das categorias profissionais, frente ao cenário de desfinanciamento da saúde.

## INTRODUÇÃO

O modelo de atenção à saúde vem sofrendo modificações ao longo dos anos, passando de uma perspectiva biologicista para a proposta de uma atenção integral à saúde. A partir dessa realidade, o Sistema Único de Saúde (SUS) requer profissionais capacitados às demandas de saúde da população, com práticas voltadas às intervenções sobre os determinantes sociais do processo saúde-doença, por meio de ações de prevenção e promoção da saúde.

Especialmente a partir dos anos 2000, diversas iniciativas surgiram com o propósito de aproximar a formação em saúde das necessidades do SUS. Destacam-se nesse cenário, a publicação da Diretrizes Curriculares Nacionais a partir de 2001^1^, além de ações indutoras como o Programa Nacional de Reorientação da Formação Profissional em Saúde (Pró-Saúde) e o Programa de Educação pelo Trabalho para a Saúde (PET-Saúde)^2^. Apesar dessas iniciativas, as estratégias formuladas por grande parte dos cursos de graduação mostram-se insuficientes para superar as evidentes limitações e discrepâncias entre a formação em saúde e as necessidades do SUS^3^.

Nesse sentido, os Programas de Residência Multiprofissional em Saúde (PRMS) se apresentam como oportunidades de pós-graduação *lato sensu*, criados com o propósito de formar profissionais para o SUS, centrando o trabalho como elemento norteador da formação^4-6^. Os Programas se destinam às profissões da área da saúde, excetuando-se a profissão médica, e buscam “favorecer a inserção qualificada dos jovens profissionais da saúde no mercado de trabalho, particularmente em áreas prioritárias do SUS” (p. 2)^7^.

Em paralelo às iniciativas de mudanças na formação em saúde, o provimento e fixação de profissionais de saúde têm sido uma preocupação no Brasil, em especial no campo da Atenção Primária à Saúde (APS). Na última década, destacaram-se o Programa de Valorização da Atenção Básica (PROVAB), voltado para médicos, enfermeiros e dentistas, e o Programa Mais Médicos para o Brasil (PMM), voltado para a profissão médica, ambos com foco na provisão de profissionais para áreas remotas e vulneráveis^8^. No entanto, na contramão a todos os estímulos supracitados, avançam no Brasil políticas de austeridade fiscal que culminam no desfinanciamento do setor.

Diante desse cenário, é importante investigar a inserção profissional no SUS de egressos de PRMS, o que representaria um retorno do investimento nessa modalidade de formação à sociedade. A temática torna-se especialmente relevante pela carência de estudos nessa linha, ou que apresentem o reconhecimento da formação nos PMRS como um diferencial para a inserção no SUS. Assim, diante do cenário apresentado, o objetivo deste estudo foi investigar a inserção profissional de egressos de PMRS voltados à APS no mercado de trabalho, especialmente no SUS, bem como a identificação de possíveis fatores associados.

## MÉTODOS

Este estudo constitui-se num recorte da pesquisa intitulada “Impacto da inserção de egressos das Residências Multiprofissionais no desenvolvimento do Sistema Único de Saúde”. Trata-se de um estudo seccional realizado com egressos de PMRS voltados à APS de todo o Brasil. Adotou-se como critério de inclusão a vinculação do programa a instituições de educação superior públicas, escolas de saúde pública ou escolas de governo; estar em funcionamento no ano de 2019; e possuir algum egresso no período de 2015 a 2019.

Previamente ao envio dos convites às instituições, foi requerida a lista dos PMRS em funcionamento ao Ministério da Educação, no mês de maio de 2019, por meio da solicitação número 23480009406201916 no Sistema Eletrônico do Serviço de Informações ao Cidadão. A partir dessas informações, identificou-se os programas que apresentavam um dos seguintes termos em sua denominação: “Atenção Básica”, “Saúde da Família”, “Saúde da Família e Comunidade” ou “Atenção Primária”. Após contato prévio, 22 programas, dos 37 elegíveis, concordaram em participar da pesquisa.

O projeto e suas emendas foram apreciados pelo Comitê de Ética em Pesquisa do Hospital Universitário Onofre Lopes (CEP/HUOL), com pareceres favoráveis números 3.744.514, 3.829.247 (Emenda 1) e 3.898.156 (Emenda 2). Além disso, o projeto tramitou pelos comitês de ética em pesquisa de todas as instituições coparticipantes que possuíam este órgão, havendo 1 recusa. A Figura 1 ilustra o fluxograma geral do estudo.

**Figura 1:** Fluxograma geral do estudo.

Após a apresentação das aprovações éticas pertinentes, foi solicitada a cada instituição a listagem nominal dos egressos dos respectivos programas, com seus endereços de e-mail e números de telefone. Foram excluídos do estudo alunos desistentes, desligados ou jubilados participantes dos programas no período mencionado (2015–2019).

Identificou-se, então, 1.159 registros de egressos válidos, dos quais nem todos possuíam a totalidade dos dados solicitados (endereço de e-mail e telefone). Os egressos foram convidados a participar do estudo prioritariamente por meio de correio eletrônico, com emissão de um convite e até cinco lembretes em espaço decrescente de dias entre eles, sendo o primeiro enviado após cinco dias, o segundo após quatro dias e assim sucessivamente. Nos casos em que o e-mail estivesse inválido, realizou-se uma busca na internet para obter outro endereço válido ou redes sociais do egresso. Quando não foi possível encontrar um e-mail válido, o convite foi realizado via redes sociais ou por aplicativo de mensagens nos casos em que havia um telefone celular atualizado. A coleta de dados ocorreu no período de 01 de junho de 2020 a 01 de setembro de 2020.

Para realizar esta pesquisa, seria suficiente uma amostra de 289 indivíduos dentro de um montante de 1.159 egressos, considerando um desfecho categórico, intervalo de confiança de 95% e margem de erro de 5%. Porém, obteve-se número de respondentes superior ao necessário.

Os dados foram coletados por meio de questionário *on-line* auto aplicado, veiculado por meio do aplicativo Google Forms. Para acessar o questionário, o egresso deveria concordar com a participação no estudo eletronicamente, tendo acesso prévio ao objetivo do estudo, riscos e benefícios e ao Termo de Consentimento Livre e Esclarecido. O instrumento de coleta estava organizado em questões fechadas ou mistas abordando: 1 – Informações pessoais gerais e formação na graduação; 2 – Aspectos relativos à outra pós-graduação; 3 – Inserção no mercado de trabalho; 4 – Inserção no SUS; 5 – Formação nos PRMS, incluindo as dimensões “Motivação pessoal”, “Abordagem pedagógica” e “Cenários de educação em serviço”. A matriz de critérios de avaliação proposta no item cinco passou por processo de validação prévio por meio da técnica de consenso de Delfos.

Delimitou-se como variável desfecho a proporção de profissionais inseridos no SUS e suas características (tipo de seleção, valorização da residência no processo seletivo, vínculo de trabalho, tempo despendido para a inserção, nível de atenção, atividades desenvolvidas, contribuição da residência para o trabalho e inserção face à pandemia de covid-19) e como variáveis independentes idade, gênero, cor, profissão, local de realização do programa de residência e inserção em programas de pós-graduação *stricto sensu* passada ou presente, sendo todas elas categóricas.

O teste qui-quadrado de Pearson foi utilizado para as análises bivariadas, enquanto para a análise múltipla foi utilizada a Regressão de Poisson com variância robusta. Foram incluídas no modelo variáveis independentes com valor p ≤ 0,20 na análise bivariada, permanecendo no modelo final aquelas com significância ≤ 0,05. O modelo proposto atendeu aos pressupostos de significância do *Omnibus Test*, ausência de superdispersão e ajuste do modelo. Para as análises inferenciais a variável “profissão” foi recategorizada em Equipe de Referência para a atenção primária (Enfermagem e Odontologia) e Equipe de Apoio (demais profissões), conforme Campos^9^. Todas as análises foram realizadas com o apoio do programa estatístico IBM SPSS, versão 20.

## RESULTADOS

Dos 1.159 egressos que se enquadravam nos critérios de elegibilidade, 13 foram descartados devido a impossibilidade de contato por um dos meios descritos. Ao todo, participaram do estudo 365 egressos de programas instalados em todas as regiões brasileiras. A proporção de respondentes por região onde cursou a residência guarda proporcionalidade com os registros recebidos (Tabela 1). Além disso, em cada região, ao menos 40% dos PMRS voltados à APS em funcionamento participaram do estudo. Do total de participantes, 80,2% declararam inserção em atividades profissionais, com vínculo ao SUS ou não. Conforme os dados disponíveis na Tabela 1, observa-se que a inserção profissional nacionalmente no SUS correspondeu a 47,9% (n = 175). Comparando a inserção no SUS entre as regiões brasileiras, constata-se maior frequência de inserção nas regiões Sudeste e Nordeste, ambas com proporção maior que a nacional (Tabela 1). Destaca-se ainda que, do total de participantes que declararam inserção profissional no SUS (n = 175), 35,4% (n = 62) também desenvolvem atividades profissionais em outros serviços (ou atividades), sem vínculo com o SUS.

**Tabela 1:** Distribuição do número de registros de egressos recebidos, do número de participantes do estudo e da inserção profissional no Sistema Único de Saúde (SUS) segundo Região brasileira de realização do programa de residência, Brasil, 2020.

	Registros		Respondentes		Inserção no SUS	
	N	%	n	%	n	%
Norte	74	6,4	21	5,8	7	33,3
Nordeste	837	72,2	263	72,1	135	51,3
Centro-Oeste	32	2,8	11	3,0	3	27,3
Sudeste	136	11,7	37	10,1	22	59,5
Sul	80	6,9	33	9,0	8	24,2
Brasil	1.159	100,0	365	100,0	175	47,9

Também se observou predominância de pessoas com idade inferior a 30 anos (54,2%), auto identificadas pelo gênero feminino (81,8%) e autodeclaradas de cor parda, preta ou indígena (56,7%) (Tabela 2). Foram indicadas 13 profissões, as mais frequentes são: Enfermagem (20,8%), Fisioterapia (13,7%) e Nutrição e Psicologia, ambas com 13,2% das respostas (Figura 2). Quanto à inserção no SUS (Tabela 2), associaram-se significativamente a este desfecho, tanto na análise bivariada como no modelo múltiplo, as profissões pertencentes à Equipe de Referência (RP ajustada = 1,87; IC95% 1,54–2,28) e a inserção na pós-graduação *stricto sensu*, esta última como fator que reduz a frequência do desfecho (RP ajustada = 0,77; IC95% 0,61–0,97). Mantidas essas duas condições mencionadas, o modelo prediz um aumento na frequência estimada do desfecho para 76%.

**Tabela 2:** Inserção profissional no Sistema Único de Saúde, conforme características dos egressos de Programas de Residência Multiprofissional em Atenção Básica, Brasil, 2020 (n = 365).

Variáveis		Total		Inserção no SUS				Não ajustado		Ajustado	
				Sim		Não					
		n	%	n	%	n	%	p^a^	RP (IC95%)^b^	p^c^	RP (IC95%)
Idade (anos)											
	< 30	198	54,2	91	46,0	107	54,0	0,408	1		
	≥ 30	167	45,8	84	50,3	83	49,7		1,09 (0,88–1,36)	–	–
Gênero											
Feminino		297	81,8	144	48,5	153	51,5	0,824	1,03 (0,78–1,37)	–	–
Masculino		66	18,2	31	47,0	35	43,0		1		
Cor											
	Branca e amarela	157	43,3	69	43,9	88	56,1	0,156	1		
	Preta, parda e indígena	206	56,7	106	51,5	100	48,5		1,17 (0,94–1,46)	–	–
Profissão											
	Equipe de referência	116	31,8	81	69,8	35	30,2	< 0,001	1,85 (1,52–2,26)	< 0,001	1,87 (1,54–2,28)
	Equipe de apoio	249	68,2	94	37,8	155	62,2		1		
Local de realização do programa										
	Capital	104	28,5	51	49,0	53	51,0	0,792	1		
	Interior	261	71,5	124	47,5	137	52,5		0,97 (0,77–1,22)	–	–
Inserção na pós-graduação *stricto sensu*											
	Sim	125	34,2	51	40,8	74	59,2	0,049	0,79 (0,62–1,00)	0,030	0,77 (0,61–0,97)
	Não	240	65,8	124	51,7	116	48,3		1		

RP: Razão de prevalência; Equipe de referência: Enfermagem e Odontologia; Equipe de apoio: demais profissões.

a Teste qui-quadrado de Pearson.

b Número 1 sinaliza a categoria de referência.

c Regressão de Poisson.

**Figura 2:** Distribuição dos respondentes egressos de Programas de Residência Multiprofissional em Atenção Básica segundo profissão, Brasil, 2020.

A Tabela 3 apresenta as características de inserção no SUS. Neste quesito, os dados revelam que apesar de, na maioria das vezes, os egressos passarem por processos formais de seleção, parte deles desenvolve as atividades profissionais no SUS por meio de vínculos profissionais instáveis, como contratos temporários (27,2%) e bolsas (8,1%). O cenário mais frequente de inserção foi o da atenção básica isoladamente (47,4%), apesar de parte dos participantes (15%) inserir-se em mais de um contexto. Há ainda um predomínio das atividades (ou ações) em atenção em saúde (84,9%) e gestão em saúde (18,6%). Apesar de amplo reconhecimento dos respondentes quanto à contribuição da formação para o trabalho (97,1%), 56,9% das respostas sinalizaram a não valorização do programa de residência no processo de seleção para o trabalho no SUS.

**Tabela 3:** Características da inserção profissional de egressos de Programas de Residência Multiprofissional em Atenção Básica no Sistema Único de Saúde (SUS), Brasil, 2020.

Variáveis		n	%
Tipo de seleção (n = 173)			
	Concurso público	73	42,2
	Processo seletivo	64	37,0
	Contratação via cooperativas	13	7,5
	Cargo comissionado	10	5,8
	Contrato	9	5,2
	Outro	4	2,3
Diferenciação de pontuação de títulos para a especialização no formato de residência (n = 167)			
	Sim	72	43,1
	Não	95	56,9
Vínculo de trabalho (n = 173)			
	Celetista	32	18,5
	Estatutário	67	38,7
	Bolsista	14	8,1
	Cargo comissionado	9	5,2
	Contrato temporário	47	27,2
	Outro	4	2,3
Tempo despendido entre a conclusão da residência e o início de uma atividade profissional no SUS (n = 170)			
	Até 6 meses	107	62,9
	De 7 meses a 1 ano	22	12,9
	Mais de 1 ano	41	24,1
Nível de atenção (n = 173)			
	Atenção básica	82	47,4
	Média complexidade	25	14,5
	Alta complexidade	27	15,6
	Atenção básica e média complexidade	10	5,8
	Atenção básica e alta complexidade	6	3,5
	Média complexidade e alta complexidade	7	4,0
	Três níveis de atenção	3	1,7
	Outro contexto	13	7,5
Caracterização da atividade profissional desempenhada (n = 172)^a^			
	Atenção à saúde	146	84,9
	Gestão em saúde	32	18,6
	Vigilância em saúde	21	12,2
	Educação em saúde	28	16,3
	Preceptoria	25	14,5
Percepção de contribuição da formação na residência para o trabalho (n = 172)			
	Sim	167	97,1
	Não	5	2,9
Inserção mediante seleção para enfrentamento da pandemia de Covid-19 (n = 172)			
	Sim	15	8,7
	Não	157	91,3

a Variável categórica exaustiva.

## DISCUSSÃO

A preocupação com a inserção e a atuação de egressos de PRMS no SUS não é recente^10,11^. Contudo, até o presente momento, são escassas as informações nesse sentido. Observou-se na literatura científica a realização de estudo com egressos de Programa de Residência Médica, porém com foco em um único programa^12^. Iniciativas de estudos nacionais envolvendo a atuação profissional de egressos foram localizadas no âmbito da graduação em saúde coletiva^13^ e Programas de pós-graduação *stricto sensu* na área da saúde^14^. Lima e Andriola^15^ reforçam que egressos de cursos de graduação ou pós-graduação assumem papel estratégico para a compreensão da efetividade social e profissional da formação vivenciada.

Nesse sentido, os resultados apresentados possuem grande contribuição para a literatura científica, especialmente considerando o momento atual, em que por um lado vivenciamos ameaças e retrocessos no SUS e por outro enfrenta-se o maior desafio dos últimos 100 anos na área da saúde em função da pandemia de covid-19.

Quando se fala em inserção de profissionais de saúde na APS, é importante destacar que essa incorporação se dá em uma das equipes previstas na Política Nacional de Atenção Básica (PNAB)^16^, que são: Equipe de Saúde da Família (ESF), Equipe de Atenção Básica (EAB), Equipe de Saúde Bucal (ESB), Núcleo Ampliado de Saúde da Família e Atenção Básica (NASF-AB) e Estratégia de Agentes Comunitários de Saúde. Enquanto as ESF, EAB e ESB oportunizam a inserção de enfermeiros e dentistas (além da categoria médica), o NASF-AB possibilita a inserção de diversas categorias profissionais como assistente social, educador físico, farmacêutico, fisioterapeuta, fonoaudiólogo, nutricionista, psicólogo, terapeuta ocupacional, médico veterinário, sanitarista, além de alguns médicos especialistas^16^.

Os NASF-AB têm como referência a metodologia de Apoio Matricial^17,18^, a qual proporciona a inserção de especialistas na APS brasileira oferecendo retaguarda e suporte técnico-pedagógico às Equipes de Referência (ESF ou EAB), podendo efetivar-se como arranjo de cuidado compartilhado e elevar a resolutividade da Atenção Primária^17,19^. Contudo, a manutenção deste dispositivo está ameaçada pela Nota Técnica nº 3/2020, que desvincula a oferta de serviços de equipe multiprofissional à tipologia de equipe NASF-AB, cessando o credenciamento de novas equipes e facultando ao gestor municipal a escolha dos profissionais da equipe multiprofissional a serem contratados, suas cargas horárias e arranjos de equipe^20^.

Nesse sentido, com base nos resultados apresentados nesta pesquisa, é possível que haja menor frequência de inserção no SUS de profissionais da Equipe de Apoio, devido às consequências da nota técnica mencionada. Assim, teme-se, como reflexo, uma diminuição da resolutividade dos serviços na APS pela redução ou ausência de especialistas.

É importante destacar que, entre os profissionais inseridos no SUS, cerca de 40% desenvolvem suas atividades laborais em vínculos instáveis. Considerando que os contratos temporários dependem de certa conjuntura político-econômica para se manterem^15^, essa instabilidade pode contribuir para um menor rendimento do trabalhador, com a respectiva fragilização do vínculo, assim como um desestímulo a futuros alunos de programas de residência, devido à incerteza de inserção no SUS.

As medidas de austeridade fiscal que o contexto político impõe nesse momento, como o teto de gastos públicos instituído por meio da Emenda Constitucional 95/2016^21^, além da ofensiva aos direitos trabalhistas e previdenciários e a ameaça da terceirização dos serviços de saúde^22^, compõem o cenário desmotivador em que se situam os achados do presente estudo.

Ademais, recentes mudanças na PNAB colocaram em risco o modelo de APS em construção no Brasil. Entre as mudanças está a recomposição das EAB que, entre outras características, admite 10h como carga horária mínima do profissional, fortalecendo uma atuação baseada no binômio queixa-conduta^23^. Em contrapartida, essa redução de carga horária fragiliza o vínculo entre o profissional e o usuário, comprometendo o conhecimento de algumas demandas que só conseguem ser identificadas por meio do vínculo afetivo.

Em paralelo ao cenário de desmonte na saúde, não se pode deixar de mencionar a reforma administrativa proposta pelo governo federal, por meio de Proposta de Emenda Constitucional 32/2020, que se aplicará aos novos servidores das três esferas da federação e que, na prática, põe em risco uma série de direitos da carreira pública^24^.

A segunda variável associada à inserção no SUS foi a inserção passada ou presente dos egressos em programas de pós-graduação *stricto sensu*, que desfavoreceu a inserção no SUS. Efetivamente, isso significa que ao estar inserido em programas de pós-graduação *stricto sensu* (mestrado ou doutorado), o egresso se distancia da inserção profissional no SUS. Este desfecho pode ocorrer por situações como opção pela dedicação exclusiva à pós-graduação, oferta de bolsas no contexto da pós-graduação e/ou investimento em uma futura carreira docente.

Estudo conduzido com egressos de cursos de graduação em Saúde Coletiva no Brasil apontou a dedicação exclusiva à pós-graduação como segundo motivo mais frequente para a não inserção no mercado de trabalho no momento da pesquisa^13^. Esse dado reitera a grande relevância de propostas de mestrados profissionais com metodologias problematizadoras para profissionais da rede, além do importante papel da educação à distância enquanto estratégia de mediação^25,26^. Nas concepções pedagógicas baseadas em metodologias problematizadoras, o ciclo de aprendizagem organiza-se a partir de situações-problemas advindas da realidade dos cursistas, contribuindo para a construção de trabalhos com foco no atendimento das necessidades locorregionais^26^. Ceccim e Pinto^27^ enfatizaram que o compromisso no enfrentamento de desigualdades regionais e sociais parte do encontro entre a formação em saúde e a rede de sistemas e serviços de saúde, pressupondo relações orgânicas.

Apesar de praticamente não haver dúvidas quanto à importância dos PRMS para o trabalho no SUS, os egressos apontam clara desvalorização dessa formação no processo de contratação para o trabalho no SUS. Isso demonstra a relevância de se construir uma Política Nacional de Residências em Saúde que preveja diretrizes de valorização dos egressos de PRMS em concursos públicos, além da qualificação e melhor respaldo do funcionamento dos próprios programas existentes^28^.

Por fim, destaca-se a inserção de uma parcela dos egressos no SUS a partir de processos seletivos voltados ao enfrentamento da pandemia de covid-19. A pandemia atingiu profundamente os serviços de saúde, demandando ampliação da força de trabalho, estruturas de atendimento e insumos para o seu enfrentamento^29^. Apesar dos anos de subfinanciamento^21^ e da ausência de uma coordenação nacional no enfrentamento da pandemia^30^, é o SUS que atende a maioria dos brasileiros e, para tanto, demanda profissionais alinhados com seus desafios, princípios e diretrizes.

Apontam-se como limitações ao presente estudo a indisponibilidade de informações pregressas relativas à inserção no SUS por parte dos respondentes, para obter a compreensão mais completa de suas trajetórias profissionais até o presente momento. Essa limitação é inerente ao desenho de estudo adotado, capaz de demonstrar apenas um corte transversal no tempo. Apesar disso, compreende-se que o estudo proporciona uma importante contribuição ao debate da formação para o SUS, uma vez que investigou a inserção profissional de egressos de PRMS voltados à APS e seus fatores associados.

A eficácia dos PRMS em APS foi claramente identificada no presente estudo ao se constatar que cerca de 80% dos egressos estudados estão inseridos no mercado de trabalho. Já quanto aos egressos inseridos no SUS, desvelou-se um cenário de instabilidade importante, assim como a flexibilidade dessa inserção, materializada pela polivalência das atividades desenvolvidas e multiplicidade de cenários de inserção. Outro ponto importante a ser destacado é a relevante percepção de contribuição dos Programas de Residência em APS para o desempenho das atividades no SUS. Demonstrou-se que quase metade dos egressos estudados estão inseridos no SUS, porém os achados deste estudo reforçam a necessidade de proposição de uma política de valorização dos PRMS no processo de recrutamento para o trabalho no SUS.

Além disso, os resultados alertam para um possível aumento da dificuldade de inserção dos profissionais da Equipe de Apoio na APS, em virtude da desobrigação do NASF-AB, tendo provável impacto na resolutividade do SUS. Ademais, a reforma administrativa em discussão pode impactar negativamente nas carreiras públicas, contribuindo para o aumento da instabilidade já existente. Estes são eventos mais recentes em um contexto maior, de desfinanciamento do setor de saúde e precarização das relações de trabalho, reforçando a relevância de realização de estudos nessa linha.

A partir do percurso trilhado, recomenda-se a continuidade dos estudos com foco na inserção profissional de egressos de PRMS, abordando outras áreas de concentração ou especialidades. A exemplo dos outros estudos com egressos citados neste texto, a pesquisa por meio digital mostrou-se uma potente ferramenta de alcance, permitindo o contato com pessoas de todo o Brasil.
